# Closed-incision negative pressure wound therapy (NPWT) in elderly patients following sacral pressure sore reconstruction

**DOI:** 10.1186/s12877-024-05526-9

**Published:** 2024-11-04

**Authors:** Ji Won Jeong, Seungkeun Lee, Jun Ho Park

**Affiliations:** 1 Department of Plastic and Reconstructive Surgery, Seoul National University College of Medicine, SMG-SNU Boramae Medical Center, 20 Boramae-ro 5-gil, Dongjak-gu, Seoul, 07061 Republic of Korea; 2 Department of Neurology, Soonchunhyang University College of Medicine, Soonchunhyang University Bucheon Hospital, Jomaru-ro 170, Bucheon, 14584 Republic of Korea

**Keywords:** Pressure sore, Pressure ulcer, Sacral sore, Negative pressure wound therapy

## Abstract

**Background:**

Pressure sores significantly affect elderly patients, with the sacrum being especially vulnerable, often due to its proximity to the anus and potential for fecal contamination. Despite preventive measures such as frequent repositioning and specialized cushions, some sores still necessitate surgical intervention. Postoperative care focuses on monitoring, hygiene, and pressure alleviation. This study assessed the efficacy of Closed-Incision Negative Pressure Wound Therapy (CI-NPWT) for postoperative wound management in patients with sacral pressure sores treated with local flaps.

**Methods:**

A retrospective analysis was performed on sacral sore patients who underwent reconstructive surgery from March 2019 through April 2023. Surgical procedures involved debridement and wound coverage using gluteal artery perforator-based fasciocutaneous flaps, followed by postoperative monitoring. Patients were grouped into conventional monitoring or NPWT management, with the latter utilizing the INFOV.A.C. The therapy unit was calibrated to 125 mmHg. Data on patient demographics, flap metrics, fluid drainage amounts, and six-month postoperative outcomes were collected, and then analyzed with SPSS Statistics.

**Results:**

In this study of 52 patients with sacral pressure sores, the NPWT group (*n* = 25) showed significantly fewer flap complications and a lower drainage volume on the seventh postoperative day (mean 17.2 cc) compared to the conventional dressing group (mean 27.8 cc, *P* < 0.05). No postoperative complications were observed in the NPWT group, whereas the conventional group (*n* = 27) experienced one infection and three cases of dehiscence. Both groups achieved a 100% flap survival rate.

**Conclusion:**

Closed-incision negative pressure wound therapy (CI-NPWT) shows promise in reducing wound dehiscence and infection rates in elderly patients undergoing sacral pressure sore reconstruction with local flaps. However, further research with larger, randomized studies is needed to confirm its effectiveness as an alternative to conventional postoperative care.

Trial registration.

The study was retrospectively registered by the Institutional Review Board of Seoul Metropolitan Government-Seoul National University Boramae Medical Center (No. 20–2023–25, Date: Mar. 24 2023).

## Background

Pressure sores continue to represent a significant source of morbidity among old patients, underscoring the critical need for effective management strategies in the realm of geriatric care. An epidemiologic study by Margolis et al. highlighted that the annual period prevalence of pressure sores in patients over the age of 65 varied from 0.31% to 0.70%, with incidence rates escalating notably with age [1]. Similarly, another investigation reported a prevalence rate of 27.9% for pressure sores in individuals aged 65 years and older within municipal healthcare settings in Sweden [2]. Pressure sores often cause significant pain, and they can also become primary infection sources for life-threatening sepsis in cases of concurrent osteomyelitis [3]. In the elderly, the risk of osteomyelitis significantly increases if surgical intervention is delayed due to deteriorating general condition. It is not uncommon for old patients and their caregivers to present at outpatient clinics with sores that have been either neglected or inadequately managed, some of which may be contaminated with fecal matter. As a prevalent and chronic issue among frail or immobilized patients in hospital environments, pressure sores also impose considerable socio-economic burden on patients, their families, and caregivers [4]. The etiology of pressure sores in old patients is complex, often compounded by comorbidities such as diabetes, stroke, and dementia [5]. Therefore, a multidisciplinary approach is often warranted for the effective treatment of pressure sore in this demographic.

The causes and pathophysiology of the development of pressure sores have been extensively elucidated in the literature. Sustained compression of a body part compromises the circulation of nearby tissues and causes direct deformation damage, eventually leading to a breach in the skin barrier or causing skin necrosis [1]. Shearing forces are also known to be one of the key factors for the development of pressure sores [6]. In terms of sore site, the sacrum is one of the areas prone to chronic pressure sores [7]. This is especially the case for paraplegic patients or those suffering from debilitating medical illnesses, who are obliged to remain in the supine position for most of their days [5]. Due to the proximity of the sacrum to the anus, this area is very prone to fecal contamination, leading to prolonged and cumbersome healing especially in patients with urinary or fecal incontinence [8].

The gold standard preventive measure for pressure sores is frequent repositioning [9]. Pressure sores can still develop in meticulous care, however, because not all caregivers are as determined to provide such labor-intensive service, especially during the nighttime when they and patients are asleep. An armamentarium of products that aim to reduce the direct pressure exerted on protruded areas of the body has been introduced, such as static air support surfaces [10] and donut cushions [11]. Nonetheless, these supplementary items sometimes pose undue stress to the patients and a single measure is often inadequate for completely preventing pressure sores [12]. Mild pressure sores with superficial skin defects can be managed with repeated dressing changes and frequent repositioning. On the other hand, moderate to severe cases of grades III or IV generally require some kind of surgical intervention for complete recovery [7]. Thanks to the ample supply of subcutaneous tissue, muscle and skin at the buttocks, many different types of flaps for the reconstruction of pressure sores in this region have been described [13] [14] [15]. Postoperative care is important for flap survival and eventual healing of the wound and mainly includes regular wound status monitoring, maintenance of hygiene, and avoidance of direct pressure.

Negative pressure wound therapy (NPWT) is a widely used mode of dressing for the management of chronic wounds and is known to hasten healing by decreasing wound edema, removing exudate, and increasing blood flow [16]. NPWT can be applied to various wound types in many different parts of the body. Closed-incision negative pressure wound therapy (CI-NPWT) serves as an effective means of postoperative wound dressing, especially in a population with an increased risk of surgical site infection [17]. In this study, we evaluated the efficacy of CI-NPWT in the management of surgical wounds in patients with sacral pressure sores treated with local flaps.

## Methods

This study was approved by the institutional review board (IRB No. 20–2023–25) of Seoul Metropolitan Government—Seoul National University, Boramae Medical Center, Seoul, Republic of Korea. We retrospectively reviewed sacral pressure sore patients treated with local transposition flaps in conjunction with NPWT. In adherence to the Declaration of Helsinki and its subsequent amendments, all participants provided written informed consent. Eligibility was granted to those who underwent flap procedures for sacral pressure sores between March 2019 and April 2023. This was a single-center study, and a single attending plastic surgeon (J.H. Park) performed all procedures with a standard protocol.

A comprehensive assessment of each patient's general condition was undertaken in collaboration with the anesthesiology department. Patients with an ASA score of 4 or above were deemed unsuitable for surgical intervention due to elevated risks, and conservative treatment modalities were considered. Conversely, in patients with an ASA score of less than 4, surgical intervention was undertaken under general anesthesia.

The operation procedure was as follows. Debridement with a Bovie ablator and Metzenbaum scissors was performed 1 to 2 weeks prior to flap coverage for induction of granulation tissue. Subsequently, the wounds were covered with gluteal artery perforator based fasciocutaneous flaps. Given the senior author’s extensive experience with pressure sores, meticulous care was taken to avoid excessive tension on the flaps at their pivot points. The flaps were manipulated so that they covered any exposed bone or ligament and were secured to adjacent viable tissues with sutures. Daily postoperative evaluations of color, temperature, capillary refill, and Doppler ultrasonography findings were performed on all flaps. All patients continued their hospital stay until the senior surgeon, J.H. Park, assessed them as medically and surgically fit for discharge.

**Fig. 1:**
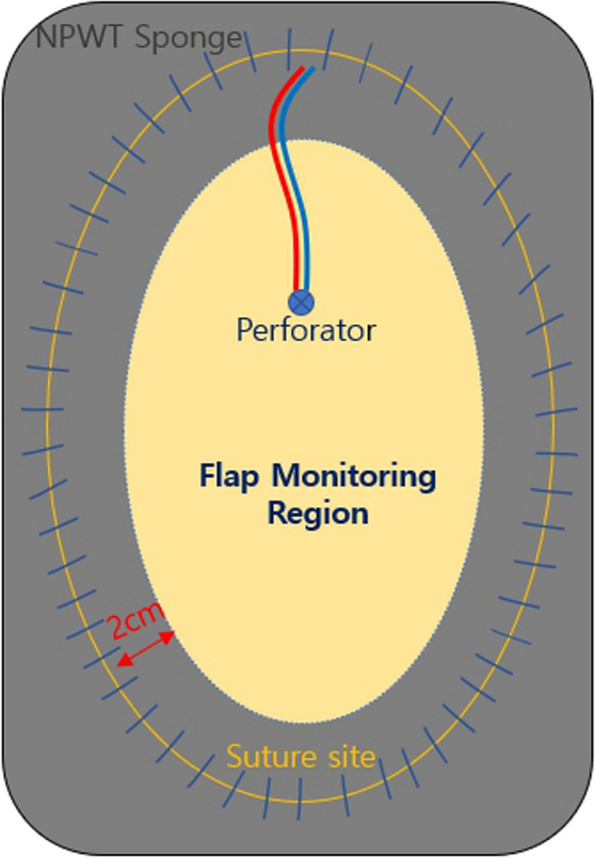
Schematic diagram of the closed-incision NPWT system for postoperative flap management

A subset of patients was allocated to the conventional monitoring group and managed with standard manual dressing and monitoring. Due to the limited supply of NPWT equipment within the ward, patients were managed with conventional dressing whenever NPWT was unavailable. Using sterile surgical gloves, the operation sites were cleansed with saline-soaked gauze and loosely covered with foam dressing material. In the NPWT management group, a black NPWT sponge (V.A.C. Granufoam, KCI, now part of 3 M Company, San Antonio, TX, USA) was positioned at the flap wound margin immediately after operation. The NPWT device (INFOV.A.C. Therapy Unit, KCI, now part of 3 M Company, San Antonio, TX, USA) was calibrated to 125 mmHg in continuous mode. To enable serial flap monitoring, a large window spanning the entire flap was created, ensuring that the sponge did not cover the flap more than 2 cm inward from the suture site (Fig. 1). Daily monitoring was conducted through this transparent window. NPWT devices were disconnected on the postoperative seventh day and transition to conventional dressing was undertaken henceforth.

The age, initial defect size, follow up period, flap surface area and operation time were recorded for each patient. The amounts of fluid collected from Jackson-Pratt or Hemovac drains were recorded on the 4th, 7th, 10th, and 14th postoperative days. Surgical outcomes including flap survival and complications were assessed six months postoperatively at outpatient clinic visits.

Statistical analyses were performed with SPSS Statistics for Windows, version 26.0 (IBM Corp., Armonk, NY, USA). Categorical variables are represented as percentages, while continuous variables are represented as the mean ± standard deviation (SD) in those following a normal distribution and as the median in those following a nonnormal distribution. The Mann–Whitney U test was employed for the analysis of continuous variables, and Fisher’s exact test was employed for categorical variables. P values of less than 0.05 were considered indicative of statistical significance.

## Results

This study involved 52 patients with sacral pressure sores treated via reconstructive surgery. Of these, 25 patients were assigned to the NPWT group, while 27 received conventional dressing postoperatively. Patient characteristics are summarized in Table 1. The mean age of patients in the NPWT group was 72.1 years, with an average follow-up period of 6.4 months, whereas patients in the conventional dressing group averaged 70.3 years of age with a 6.2-month follow-up period. Mean defect sizes and flap surface areas were 36.7 cm^2^ and 62.9 cm^2^ for the NPWT group and 34.4 cm^2^ and 65.3 cm^2^ for the conventional group, respectively. Mean operative times were similar between groups at 88.0 min for the NPWT group and 86.5 min for the conventional group, with no statistically significant difference noted.

**Table 1:** Characteristics and surgical outcomes of the CI-NPWT group and conventional dressing group

	**NPWT group**	**Conventional group**	**P value**
Number of patients	25	27	
Age, years, mean ± SD	72.1 ± 11.1	70.3 ± 13.3	0.639
Defect size, cm^2^, mean ± SD	36.7 ± 19.6	34.4 ± 17.7	0.582
Follow up, months, mean ± SD	6.4 ± 0.5	6.2 ± 0.4	0.882
Flap surface, cm^2^, mean ± SD	62.9 ± 7.6	65.3 ± 6.8	0.744
Operation time, min, mean ± SD	88.0 ± 23.7	86.5 ± 17.3	0.807
Amount of drainage, cc, mean ± SD	17.2 ± 13.0	27.8 ± 19.8	0.027
Flap Complications	0	4	0.014
Infection	0	1	
Hematoma	0	0	
Seroma	0	0	
Dehiscence	0	3	
Flap necrosis	0	0	

**Fig. 2:**
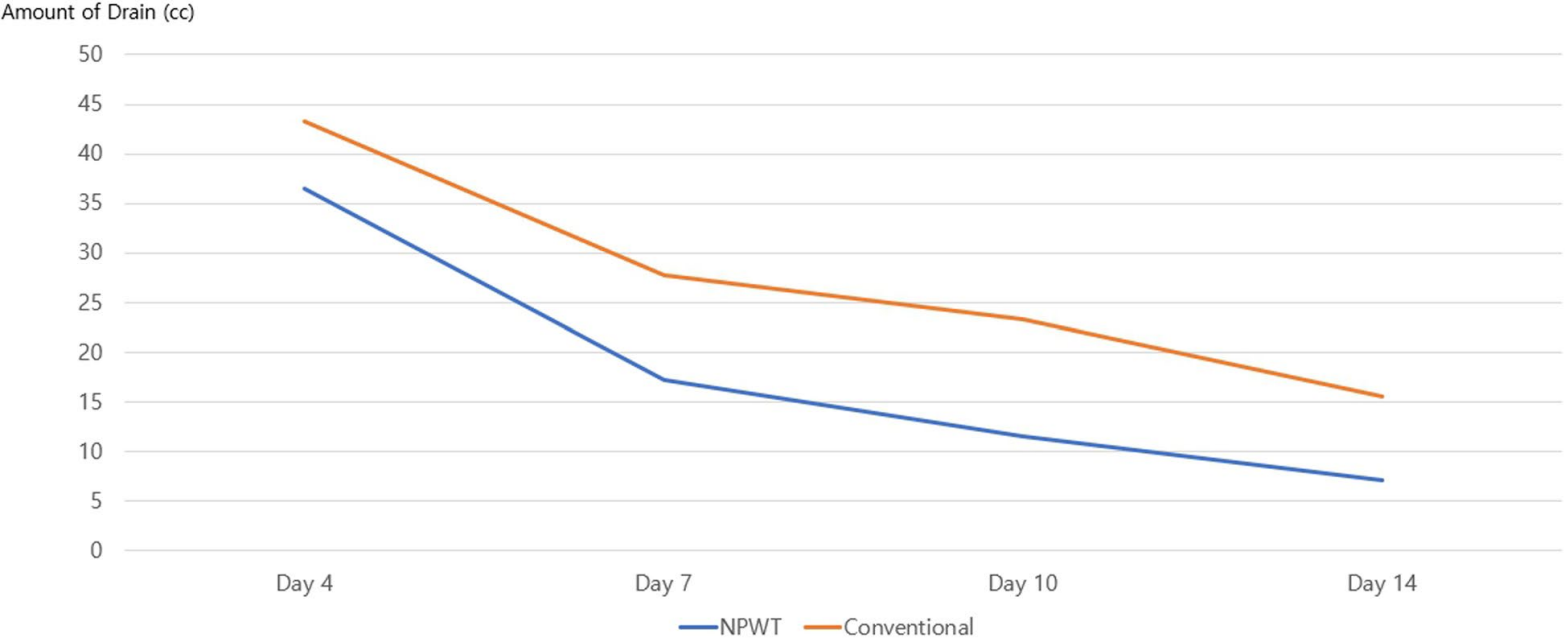
Changes in the amount of postoperative drainage by day

Both groups demonstrated a 100% flap survival rate. However, the NPWT group exhibited significantly fewer flap complications and a reduced drainage volume on the seventh postoperative day (mean 17.2 cc) compared to the conventional dressing group (mean 27.8 cc,* P* < 0.05) (Fig. 2). These trends continued postoperatively, as the NPWT group maintained lower drainage volumes even after NPWT equipment was discontinued at seven days. Within the follow-up period, the conventional group experienced one wound infection and three instances of dehiscence, whereas the NPWT group did not exhibit any postoperative complications.

**Fig. 3:**
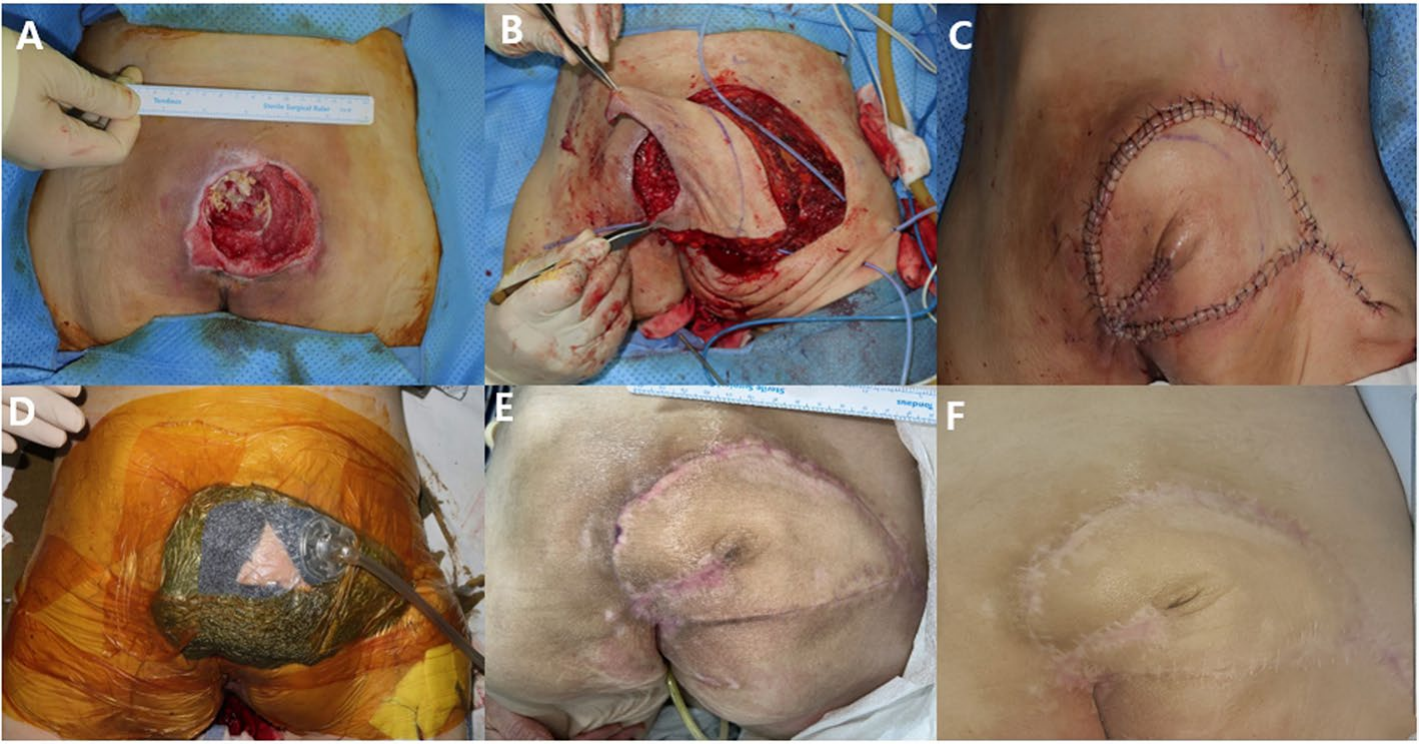
**A** A 78-year-old female presented with a grade IV pressure sore. **B** Superior gluteal artery perforator-based flap elevation with subfascial dissection. **C** Transposition of the elevated flap and inset on the defect area. **D** CI-NPWT application with a monitoring window. **E** Postoperative photographic findings 2 months, and (**F**) 6 months after the operation

**Fig. 4:**
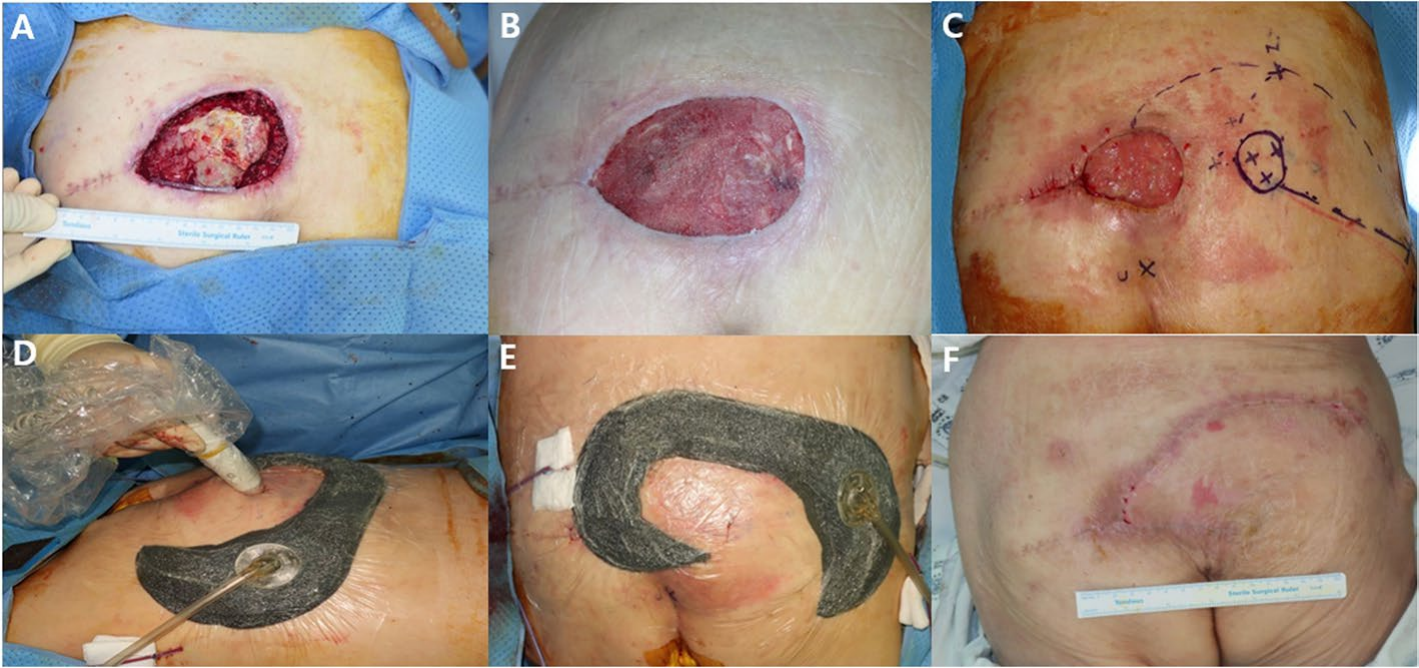
**A** A 78-year-old female presented with a grade IV pressure sore. **B** Photographic finding 2 weeks after serial debridement and NPWT application. **C** Flap design after wound dehiscence of initial primary closure. **D** Flap monitoring with Doppler probe immediately after flap operation. **E** Photographic finding after fasciocutaneous rotation flap and CI-NPWT application. **F** Photographic finding 1 month after operation

Figures 3 and 4 illustrate the healing progress, showing preoperative, intraoperative, and postoperative stages in patients treated with NPWT. Wound healing in both depicted cases proceeded without complications.

## Discussion

The significance of pressure sores is growing with the aging of the general population. Primary preventive measures include frequent repositioning, maintenance of good nutrition, and the use of specialized bedding and cushions. Pressure sores can still develop despite primary prevention, and thus early detection in conjunction with secondary prevention are crucial for patients’ well-being. Secondary preventive measures include the use of ointment and foam dressing materials to accommodate secondary healing. Pressure ulcers of grade III and IV, on the other hand, often necessitate surgical treatment with local flaps. Postoperative management of local flaps for pressure sores is critical for the survival of such flaps. There must be no pressure or tension exerted on the surgical wounds, and maintenance of hygiene hinders the chance of infection. Patients are often encouraged to stay in prone or lateral decubitus positions for at least two weeks following their operation. The complication rate after local flap operation on sacral pressure sore has been reported to be as high as 27.5% [18], with wound dehiscence being a common type of complication. Repeated wound revision generally involves trimming of nonviable tissue and reapproximation with sutures, depriving the sacrococcygeal area of reserve of healthy tissue. This can, in turn, result in increased skin tension and a higher likelihood of secondary or tertiary dehiscence, precipitating a vicious cycle. Thus, prevention of wound dehiscence is of vital importance for the long-term well-being of patients and minimization of their hospital stay.

Ever since Argenta and colleagues introduced the VAC device in 1997 [19], it has been used to promote wound healing in a variety of medical conditions. Normandin and colleagues described the mechanism of NPWT in their review paper [20]. The four dominant mechanisms of NPWT are macrodeformation, drainage of fluids, stabilization of the wound environment, and microdeformation. Macrodeformation refers to the physical wound contraction rendered by the subatmospheric pressure that the device exerts on the wound. The sponge can shrink as much as 80% in the negative pressure setting, effectively bringing the firmly attached tissues into vicinity [16]. The removal of extracellular fluids alleviates edema and cellular compression that would otherwise have inhibited the proliferative cellular response [20]. With the use of NPWT, less fluid accumulates around small blood vessels and the wound is more effectively perfused with blood. Moreover, toxic extracellular materials such as TNF-α and matrix metalloproteinase (MMP) are constantly removed from the wound, allowing for more rapid recovery [21]. NPWT also stabilizes the wound environment by preventing bacterial colonization and reducing heat loss. Polyurethane drape is impermeable to microorganisms and protein, making it more difficult for biofilms to develop. Meanwhile, the semiocclusive membrane traps water vapor and heat, keeping the wound moist and warm. Microdeformation refers to the ability of NPWT to regulate cellular response and promote the formation of granulation tissue via mechanotransduction. According to Saxena and colleagues, negative pressure disrupts integrin bridges and releases intracellular messengers that ultimately induce genetic transcription for cell proliferation [22]. The literature also describes the proliferation of fibroblasts and inhibition of apoptosis as components of the microdeformation theory [23] [24]. Many papers have been published focusing on the superiority of NPWT over conventional dressing in wound healing. Blume and colleagues reported that complete wound epithelialization was achieved in 43.2% of diabetic foot ulcer patients treated with NPWT compared to 28.9% in patients treated with conventional moist wound therapy [25]. Suissa and colleagues reported in their meta-analysis that NPWT significantly reduced the sizes of chronic wounds compared to standard wound care, and that the healing period was significantly shorter for the former group [26]. It is also known that wounds treated with VAC become suited for skin graft earlier than wounds managed with conventional dressing [27]. In terms of pressure sores, Schwien and colleagues demonstrated that patients managed with NPWT had fewer hospitalizations than their counterparts who were treated with standard moist dressing [28].

Closed-incision NPWT is a relatively novel approach for surgical wound management. Condé-Green and colleagues reported in their retrospective chart review that abdominal wall reconstruction wounds treated with CI-NPWT exhibited a significantly lower rate of complications than those treated with conventional gauze dressings (22 vs. 65.9%, respectively) [29]. Such complications included wound dehiscence, infection, skin and fat necrosis, seroma and hernia recurrence. Likewise, Stannard and colleagues demonstrated fewer cases of infection and dehiscence after high-risk lower extremity trauma in the CI-NPWT group [30]. The efficiency of flap monitoring with CI-NPWT has also been reported in previous literature [31] [32]. Compared to conventional dressing, there was no significant difference in overall flap survival or the diameters of flap perforator vessels. On the other hand, the mean monitoring time per flap was significantly reduced in the CI-NPWT groups, alleviating the workload of medical staff [32]. Conventional dressing groups necessitated more frequent dressing changes and resulted in higher overall costs compared to CI-NPWT groups [31]. The authors further speculated that frequent manual dressing change could increase the risk of wound infection. The ease of wound monitoring and reduced labor of medical staff, along with the possibly lowered risk of iatrogenic wound infection are all applicable to the subjects of this study. The superiority of CI- NPWT was also evident in scalp reconstruction with local flaps and skin grafts, where the authors demonstrated a higher percentage of mean graft take in patients treated with CI-NPWT [33].

In this study, we showed that CI-NPWT application in patients treated with local flaps for sacral pressure sores effectively reduces the rate of wound dehiscence and the subsequent need for revision. There could be several explanations for this finding. First, NPWT effectively reduces the risk of fecal contamination of wounds compared to conventional dressing methods. Airtight wound sealing is essential for the maintenance of negative pressure of proper intensity and therefore there is almost no possibility of solid or liquid material seeping into the dressing. In case of a breach in airtight sealing, the alarm set off by the NPWT equipment guarantees prompt intervention and resealing, precluding unnoticed wound contamination. Aseptic dressing with NPWT equipment every 3 ~ 5 days is expected to be safer than conventional dressing once or even twice a day with regards to wound infection. Indeed, Hyldig and colleagues demonstrated the superiority of CI-NPWT over standard dressing in terms of wound infection in their randomized controlled trial with obese women undergoing Caesarian section [34]. Yutaro et al. detected bacterial infection in all wounds with early dehiscence in patients treated with pressure ulcer surgery [35], which in turn emphasizes the role of wound hygiene in preventing dehiscence as well as infection. In our study, one patient in the conventional dressing group experienced wound infection while no such case was found in the CI-NPWT group.

The increased degree of tissue vascularization provided by NPWT could also be one of the factors for the expedited recovery and wound stability in pressure sore patients. Considering the suboptimal general condition and commonly encountered malnutrition among these patients [36], such enhancement in the niche of wound healing is indispensable. The results of our study are consistent with those in previous literature indicating a positive effect of CI-NPWT on wounds that may otherwise have been prone to infection or other complications [37] [38]. Enhanced angiogenesis induced by NPWT is thought to hinder the chance of infection, due to the robust supply of blood and its accompanying white blood cells to the area of tissue healing. Previous literature points to the positive effect of NPWT on flap perfusion via resolution of venous congestion [39] [40]. Since flap perfusion is critically important for wound stability, this could be another protective factor of NPWT against dehiscence.

Another strength of NPWT is its ability to constantly remove excessive tissue exudate and thus reduce the load on surgical drains. Seroma or hematoma are commonly encountered complications that can occur even when drains are in place. This is especially the case when the rate of extracellular fluid generation or capillary blood oozing exceeds the capacity of the drains. Thus, a double-drainage system consisting of CI-NPWT and surgical drains would be more effective in preventing fluid collection. Considering the increased likelihood of wound infection due to seroma serving as a culture medium, this double-drainage system could be one of the explanations for the lowered risk of infection. Moreover, the shear volume effect of fluid collection can cause wound disruption. It could be inferred that CI-NPWT lowers the risk of wound dehiscence by minimizing such potential fluid accumulation. In our study, the CI-NPWT group exhibited smaller drainage amounts on the seventh postoperative day than the conventional dressing group. Therefore, it is likely that patients treated with CI-NPWT had drains removed earlier than their counterparts. Dragu and colleagues have demonstrated such findings in their study of NPWT dressing in abdominal dermolipectomy patients [41]. Earlier drain removal in turn shortens the period of foreign body residence in vitro, another potential source of infection.

Patient compliance is another important factor in the ultimate outcome of pressure sore operations. It is often the case that pressure sore patients find it difficult to follow their physician’s instructions on position changing and maintaining hygiene. Direct pressure to the operation site after local flap placement on the sacrum can be hazardous, and patients are strongly advised to maintain the prone or lateral decubitus position for at least two weeks. In the senior author’s experience, however, many patients are found resting in the supine position during rounds. CI-NPWT can also be helpful in such cases, since the default negative pressure exerted on the wound at least partially compensates for the positive normal pressure from the bed. Although not covered in this study, NPWT holds superiority over conventional dressing even in the out-of-hospital setting, as there is no need for patients to change dressing by themselves in the former group.

In the realm of geriatric care, the advantages CI-NPWT in managing sacral sores post-flap surgeries are notably significant. Elderly patients and their caregivers often struggle with adhering to the aseptic techniques required for manual dressing changes, leading to an increased risk of wound contamination. Conversely, NPWT is typically administered by healthcare professionals, offering a more dependable approach to maintaining wound cleanliness. The principle of enhanced tissue vascularization through CI-NPWT is particularly relevant to the elderly population, in whom wound healing is naturally decelerated due to age-related physiological changes. The augmented blood supply facilitated by NPWT can significantly accelerate the healing process in older individuals. Additionally, the reduced necessity for frequent positional changes with NPWT use presents a substantial benefit for elderly patients, whose compliance may not be as feasible as that of younger individuals. Furthermore, CI-NPWT aims to minimize the incidence of wound dehiscence following flap surgeries, thereby diminishing the potential need for revision surgery under general anesthesia. Given the heightened risks associated with repeated anesthesia in elderly patients with pre-existing chronic conditions and multiple comorbidities, CI-NPWT emerges as a particularly advantageous treatment strategy.

This study has several limitations that warrant caution in interpreting the findings. First, the sample size was small and the allocation non-random, as patients were assigned to conventional dressing when NPWT equipment was unavailable, introducing potential selection bias. The short follow-up period may also limit our ability to assess long-term complications or the durability of NPWT benefits. Additionally, we did not account for financial factors, including the affordability of specialized dressing materials, which could impact consistency in conventional care. Variations in patient history, such as previous sacral surgeries, as well as uncontrolled factors like preoperative bacterial infections and general medical conditions, represent further potential confounders. Finally, cost-effectiveness was not evaluated, though this is critical for broader clinical application. Future studies with larger, randomized samples, extended follow-up, and cost analyses are needed to confirm and expand on these findings, and newer NPWT modalities with intermittent wound cleansing should also be explored for enhanced outcomes.

## Conclusion

Closed-incision negative pressure wound therapy (CI-NPWT) may improve outcomes by reducing wound dehiscence and infection in elderly patients with sacral pressure sores treated with local flaps. While these results are encouraging, further research with larger, randomized studies is needed to confirm CI-NPWT’s efficacy. With additional validation, CI-NPWT could become an effective alternative to conventional monitoring in postoperative care for this population.

## Data Availability

The datasets generated and/or analysed during the current study are not publicly available as individual privacy could be compromised but are available from the corresponding author on reasonable request.

## Abbreviations

CI-NPWT: Closed-incision negative pressure wound therapy
VAC: Vacuum assisted wound closure
ASA score: American Society of Anesthesiologists score
NPWTi-d: Negative pressure wound therapy with instillation
